# snakePipes: facilitating flexible, scalable and integrative epigenomic analysis

**DOI:** 10.1093/bioinformatics/btz436

**Published:** 2019-05-27

**Authors:** Vivek Bhardwaj, Steffen Heyne, Katarzyna Sikora, Leily Rabbani, Michael Rauer, Fabian Kilpert, Andreas S Richter, Devon P Ryan, Thomas Manke

**Affiliations:** 1 Max Planck Institute of Immunobiology and Epigenetics, 79108 Freiburg, Germany; 2 Faculty of Biology, University of Freiburg, 79104 Freiburg, Germany; 3 Institutes of Neurogenetics & Cardiogenetics, University of Lübeck, 23562 Lübeck, Germany; 4 Genedata AG, 4053 Basel, Switzerland

## Abstract

**Summary:**

Due to the rapidly increasing scale and diversity of epigenomic data, modular and scalable analysis workflows are of wide interest. Here we present snakePipes, a workflow package for processing and downstream analysis of data from common epigenomic assays: ChIP-seq, RNA-seq, Bisulfite-seq, ATAC-seq, Hi-C and single-cell RNA-seq. snakePipes enables users to assemble variants of each workflow and to easily install and upgrade the underlying tools, via its simple command-line wrappers and yaml files.

**Availability and implementation:**

snakePipes can be installed via conda: `conda install -c mpi-ie -c bioconda -c conda-forge snakePipes’. Source code (https://github.com/maxplanck-ie/snakepipes) and documentation (https://snakepipes.readthedocs.io/en/latest/) are available online.

**Supplementary information:**

Supplementary data are available at *Bioinformatics* online.

## 1 Introduction

The decreasing price of sequencing and increasing multiplexing ability has allowed researcher to easily produce large datasets. To understand genetic and epigenetic regulation, researchers routinely perform multiple assays, such as RNA-seq and Bisulfite-seq in the same project, necessitating scalable data processing workflows. Since exploratory studies demand more flexibility in data processing, and standards evolve rapidly, conventional rigid pipelines become quickly outdated. Computational frameworks, such as Galaxy (Goecks *et al.*, 2010), Nextflow (Di Tommaso *et al.*, 2017) and snakemake (Köster and Rahmann, 2012) address this issue to some extent by allowing users to create their own workflows, or adopt workflows from public repositories. However, these frameworks are still challenging for novice users, as they require training in their specific programing language or syntax and assembling workflows themselves. This leads to a conundrum, how can we offer the flexibility of assembling and upgrading analyses workflows to the novice users, while still keeping them scalable and reproducible?

We developed snakePipes to address this issue. snakePipes provides a set of best-practices workflows for processing, quality control and downstream analysis of data from the most common assays used in epigenomic studies: ChIP-seq, RNA-seq, whole-genome bisulfite-seq (WGBS), ATAC-seq, Hi-C and single-cell RNA-seq (Supplementary Fig. S1a; Supplementary Methods). However, unlike conventional pipelines, workflows in snakePipes are based on a repository of modular rules, such that multiple variations of each workflow can be assembled on-the-fly by changing the parameters on their command-line wrappers. This novel approach allows novice users to perform exploratory analysis in a reproducible way without manually assembling workflows.

## 2 Implementation

**Fig. 1.**
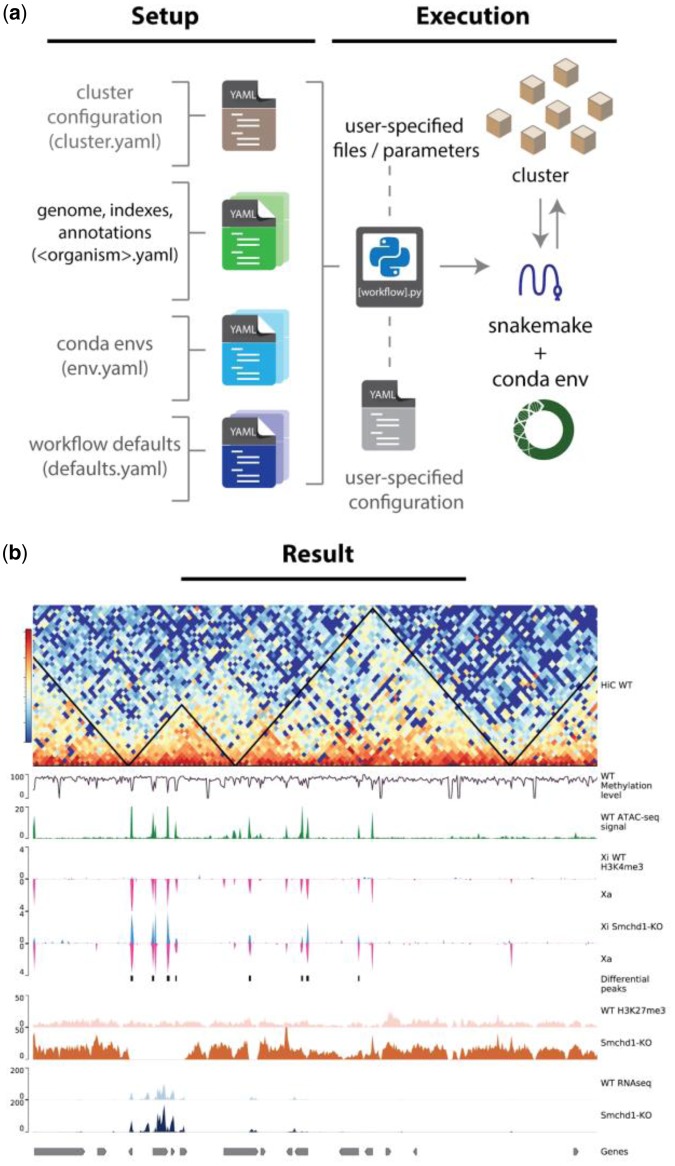
Setup, execution and results from snakePipes. (**a**) All configurable parameters for snakepipes are defined as YAML files during setup. However, most parameters can be overwritten during execution by providing another YAML file, adding flexibility to the analysis. (**b**) Output of HiC (track 1), WGBS (track 2), ATAC-seq (track 3), allele-specific ChIP-seq (tracks 3–7) and RNA-seq (tracks 8–9) workflows, plotted using pyGenomeTracks (Ramírez *et al.*, 2018)

snakePipes employs snakemake (Köster and Rahmann, 2012) as its core workflow language, which benefits from easy readability of the code, widespread adoption and scalability to most clusters and cloud platforms. snakePipes also makes use of conda environments and the bioconda platform (Grüning *et al.*, 2018), which allows hassle-free installation and upgrade of known-compatible and known-functional tools (Fig. 1a;Supplementary Methods). Conda environments alleviate the need to manually manage tools or have administrator permissions.

snakePipes’ modular architecture allows various tools and resources to be shared between workflows, simplifying data integration since data from multiple assays are processed using identical tool versions. Genome annotations and indices are shared by all workflows, and can also be generated directly via snakePipes, facilitating easy setup as well as integrative analysis. Finally, all workflows in snakePipes calculate extensive quality control metrics and produce reports using multiQC (Ewels *et al.*, 2016) and R, that inform the user of processing and analysis results.

Apart from conventional processing steps such as mapping and peak calling, workflows in snakePipes also include various downstream analyses. All workflows (except scRNA-seq workflow) optionally accept a sample information (tab-separated) file that can be used to define groups of sample. This allows comparative analysis, such as differential expression (RNA-seq), differential peak calling (ChIP-Seq), differential accessibility (ATAC-seq) and differential methylation (WGBS). Complex design formulas are supported using additional columns of the sample sheet. The HiC workflow uses sample information to merge groups and can perform TAD calling with parameters adapted to the resolution of the produced matrix [using HiCExplorer (Ramírez *et al.*, 2018)]. Most workflows also allow allele-specific processing of data via SNPSplit (Krueger and Andrews, 2016) where a single or dual-hybrid genome can be created on-the-fly using the ‘allelic-mapping’ mode and a Variant Call Format file (Danecek *et al.*, 2011). Further downstream analysis, such as allele-specific differential expression can be performed automatically. This preliminary analysis, combined with visualization-ready BED and bigWig files, allows users to quickly interpret their data (Fig. 1b). Our comparison with other recently released workflows and pipelines suggests that snakePipes offers the most extensive processing and analysis options under a single package. Further, it compares equally well to the other available alternatives in terms of installation, ease of use and scalability (Supplementary Table S1).

## 3 Application

To demonstrate how snakePipes can simplify analysis of data from multiple epigenomic assays, we processed data from a study of the mammalian X-chromosome (Wang *et al.*, 2018). The knock-out of Smchd1 in mouse neural progenitor cells affects the X-chromosome organization and leads to a loss of H3K27me3 domains, gain of H3K4me3, along with de-repression of genes on the inactive X-chromosome. These changes are apparent directly from the snakePipes output (Fig. 1b;Supplementary Fig. S1b and c). We further combined these results with those obtained from online ATAC-seq (Giorgetti *et al.*, 2016) and WGBS data (GSE101090) processed via snakePipes, and find that these de-repressed genes have a higher open chromatin signature compared to the downregulated or unchanged genes (Supplementary Fig. S1d). These genes also show a methylation status similar to the downregulated but lower than unchanged genes (Supplementary Fig. S1e), corroborating previous (Schübeler, 2015) and recent (Lea *et al.*, 2018) links between promoter CpG methylation and gene repression.

## 4 Conclusion

In summary, snakePipes simplifies the analysis of large-scale epigenomic studies by allowing fast and reproducible processing of data from several assays. While further downstream analysis would still be required to integrate the results depending upon biological questions, snakePipes’ outputs allow biologists to quickly interpret and understand their results, facilitating integrative analysis.

## Supplementary Material

btz436_Supplementary_Data

## References

[btz436-B3] Danecek P. et al (2011) The variant call format and VCFtools. Bioinformatics, 27, 2156–2158. 21653522 10.1093/bioinformatics/btr330 PMC3137218

[btz436-B4] Di Tommaso P. et al (2017) Nextflow enables reproducible computational workflows. Nat. Biotechnol., 35, 316–319. 28398311 10.1038/nbt.3820

[btz436-B5] Ewels P. et al (2016) MultiQC: summarize analysis results for multiple tools and samples in a single report. Bioinformatics, 32, 3047–3048. 27312411 10.1093/bioinformatics/btw354 PMC5039924

[btz436-B7] Giorgetti L. et al (2016) Structural organization of the inactive X chromosome in the mouse. Nature, 535, 575–579. 27437574 10.1038/nature18589 PMC5443622

[btz436-B8] Goecks J. et al (2010) Galaxy: a comprehensive approach for supporting accessible, reproducible, and transparent computational research in the life sciences. Genome Biol., 11, R86. 20738864 10.1186/gb-2010-11-8-r86 PMC2945788

[btz436-B9] Grüning B. et al (2018) Bioconda: sustainable and comprehensive software distribution for the life sciences. Nat. Methods, 15, 475–476. 29967506 10.1038/s41592-018-0046-7 PMC11070151

[btz436-B11] Köster J., Rahmann S. (2012) Snakemake–a scalable bioinformatics workflow engine. Bioinformatics, 28, 2520–2522. 22908215 10.1093/bioinformatics/bts480

[btz436-B12] Krueger F., Andrews S.R. (2016) SNPsplit: allele-specific splitting of alignments between genomes with known SNP genotypes. F1000Res., 5, 1479. 27429743 10.12688/f1000research.9037.1 PMC4934512

[btz436-B13] Lea A.J. et al (2018) Genome-wide quantification of the effects of DNA methylation on human gene regulation. Elife, 7, e37513. 30575519 10.7554/eLife.37513 PMC6303109

[btz436-B14] Ramírez F. et al (2018) High-resolution TADs reveal DNA sequences underlying genome organization in flies. Nat. Commun., 9, 189. 29335486 10.1038/s41467-017-02525-w PMC5768762

[btz436-B16] Schübeler D. (2015) Function and information content of DNA methylation. Nature, 517, 321–326. 25592537 10.1038/nature14192

[btz436-B17] Wang C.-Y. et al (2018) SMCHD1 merges chromosome compartments and assists formation of super-structures on the inactive X. Cell, 174, 406–421.e25. 29887375 10.1016/j.cell.2018.05.007 PMC6475921

