# Supplementary material for: snakePipes: facilitating flexible, scalable and integrative epigenomic analysis
Source: Bioinformatics. 2019 May 27;35(22):4757–9. doi: 10.1093/bioinformatics/btz436 (PMC6853707; doi:10.1093/bioinformatics/btz436)
Supplement: btz436_Supplementary_Data [file btz436_supplementary_data.zip › btz436-suppl_data/Supplementary_Material.pdf]

Supplementary Material

Supplementary Fig. 1

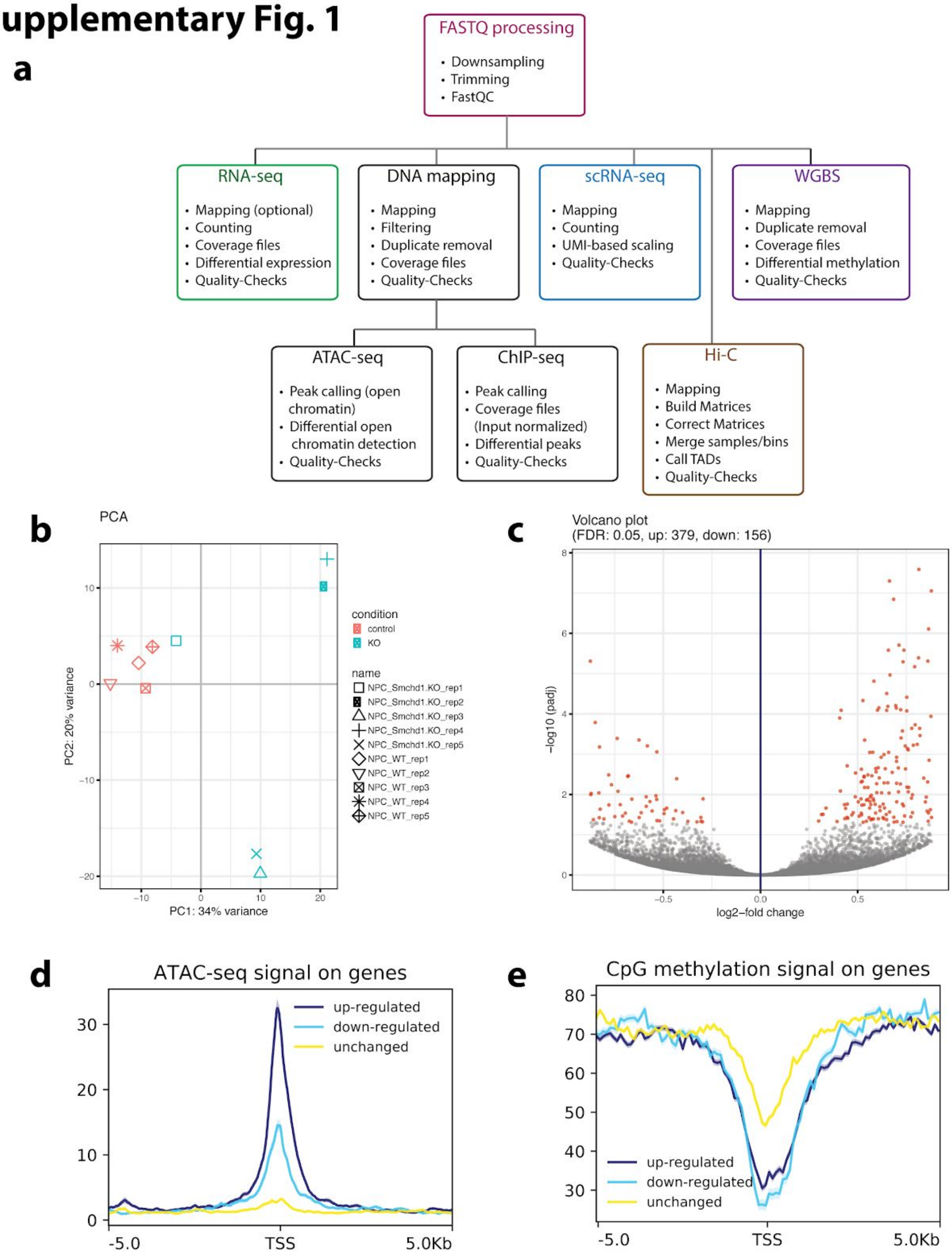

Supplementary Fig. 1. a. Workflows in snakePipes. *FASTQ files provided by the user are first processed by common steps, followed by one or more sample-specific workflows. Outputs from DNA mapping workflow can be further used as input for the ChIP-seq and ATAC-seq workflows. Only general processing steps are listed and each workflow includes workflow-specific quality-checks.* ***B.*** *Analysis of de-repressed genes upon Schmd1 knock-out. PCA output from snakepipes suggested that one knock-out sample (replicate1) behaves differently. This sample was later revealed to be the XO clone which lost it's inactive X chromosome. The sample was removed for DESeq2 analysis and the workflow was re-run.* ***C.*** *Volcano plot for DESeq2 output from snakePipes (knock-out replicate 1 excluded), shows an increase in up-regulated genes, indicating de-repression upon knock-out.* ***D.*** *Wild-type ATAC-seq signal on UP, DOWN and unchanged (NONE) genes, gene lists were extracted from DESeq2 output of RNA-seq workflow and depth-normalized bigwigs from ATAC-seq workflow was used for plotting.* ***E.*** *Wild-type methylation level reported by the WGBS workflow on UP, DOWN and unchanged (NONE) genes. (TSS = Transcription Start Site)*

Supplementary Information

General architecture of snakePipes

The general architecture of snakePipes is summarized in Figure 1a. snakePipes utilizes conda and bioconda for setup and execution of workflows. All information required for workflow execution is stored in easy-to-edit YAML (Yet Another Markup Language) files. For each organism of interest, the <**organism>.yaml** file describes the location of genome fasta, (mapping) indices and annotations. This allows running different workflows on exactly the same version of genome and annotations. The conda **env.yaml** file is used to specify version number of required tools for each workflow. The required tools are then fetched and setup automatically via the conda and bioconda repositories either during workflow setup, or execution. The **defaults.yaml** file specifies reasonable default parameter for each workflow according to the best practices for the most common sequencing protocols. After installation, the location of all the YAMLs as well as the workflow rules can be revealed by the "**snakePipes info**" command. These files are used by the command line wrappers, that use snakemake to execute the workflows on the cluster. In the absence of cluster setup, workflows can also be executed

locally. Each step of a workflow is defined as a snakemake “rule”, which is executed in it’s own virtual environment, avoiding conflicts between tools. All log files, including user-supplied commands are written in the working directory, along with (optionally) a graph of executed steps. This allows users to easily reproduce and communicate their analysis in the future.

Running and testing the workflows

Comprehensive documentation for snakePipes can be found online: https://snakepipes.readthedocs.io/ and the test datasets are available on zenodo : https://zenodo.org/record/1346303 . snakePipes also provide a `***createIndices***` workflow that creates genome indices and annotations (contents of <organism>.yaml) from a user-specified genome fasta file or URL.

Cluster and cloud support

Within snakePipes, users can take full advantage of snakemake for execution of workflows to a cluster or on the cloud. A cluster.yaml file defines the command used to execute rules on a cluster, which can be modified by the user during setup. Execution via DRMAA provides better error handling, while execution via command-line cluster wrappers provide more control and customization to the user. We also provide easy-to-use scripts that use Slurm to submit jobs to the cluster, and can be used with snakePipes by users which these working on such clusters. Usage on cloud architecture is essentially identical to a local cluster and outlined in the online documentation.

Workflows in snakePipes

snakePipes provide DNA-mapping, ChIP-seq, ATAC-seq, RNA-seq, whole-genome bisulfite-seq (WGBS), HiC and single-cell RNA-seq workflows. All workflows make use of the common fastq downsampling (via seqtk (https://github.com/lh3/seqtk)) and trimming (via cutadapt (Martin, 2011) and Trim Galore! (https://www.bioinformatics.babraham.ac.uk/projects/trim_galore/)) module. All workflows also produce an interactive report using multiQC (Ewels *et al.*, 2016) that summarizes outputs from multiple workflow steps and samples.

In the **DNA-mapping** workflow, the fastq files are aligned to the genome via Bowtie2 (Langmead and Salzberg, 2012) and filtering can be performed via samtools (Li *et al.*) using user-provided parameters. Various quality-checks are performed via SamBamba (Tarasov *et al.*, 2015), Picard (McKenna *et al.*, 2010), deepTools (Ramírez *et al.*, 2016) and (optionally) qualimap (García-Alcalde *et al.*, 2012). Coverage files (bigwigs) are generated via deepTools. The output of DNA-mapping workflow can then be used for ChIP-Seq or ATAC-seq workflows. DNA-mapping workflow handles both single and paired-end files and could also be used for whole-genome alignments.

The **ChIP-seq** workflow takes information about the samples (corresponding input controls, expected broad/sharp mark) using a yaml file, and performs ChIP-specific quality-checks via deepTools. It also generates input-normalized bigwig files and performs peak calling for both sharp (via MACS2 (Feng *et al.*, 2012)) and broad (via histoneHMM (Heinig *et al.*, 2015)) marks. The **ATAC-seq** workflow takes paired-end DNA mapping output and performs quality-checks and filtering useful for ATAC-seq samples. It then performs detection of open chromatin using MACS2. Both ChIP-Seq and ATAC-Seq workflows can perform detection of differential peaks or differential open chromatin regions between groups of samples using CSAW (Lun and Smyth, 2016), if a sample sheet is provided.

The **RNA-seq** workflow can be run in "alignment" or "alignment-*free*" mode. In the alignment mode, fastq files are aligned to the genome via user-selected aligner (STAR (Dobin *et al.*, 2013) or HISAT2 (Kim *et al.*, 2017)) and high-quality primary alignments are counted via featureCounts (Liao *et al.*, 2014). In the alignment-free mode, the transcripts are directly quantified via Salmon (Patro *et al.*, 2017). Transcripts can be filtered for various features before quantification. Additionally, the "*deepTools_qc*" mode can be added, which performs various quality-checks via deepTools and produces normal and depth-normalized (RPKM) coverage files. Differential gene and transcript expression analysis could then be performed using DESeq2 (Love *et al.*, 2014), wasabi (https://github.com/COMBINE-lab/wasabi) and Sleuth (Pimentel *et al.*, 2017).

The **scRNA-seq** workflow performs mapping and counting of data obtained from the CEL-Seq2 protocol (Hashimshony *et al.*, 2016). Fastq files are first preprocessed by moving cell barcodes

and unique molecular indices (UMIs) to read headers and then mapped using STAR (Dobin et al.). Quantification is then performed per-cell by accounting for UMIs and the resulting counts corrected for Poisson sampling. A variety of quality control steps are also taken, such as computing a heatmap per well-plate of obtained transcript counts and the correlation between reads and UMIs. After the workflow is finished the resulting counts files are ready for custom downstream analysis (e.g., clustering or differential expression).

The **Hi-C** workflow performs read mapping of paired-end HiC data using BWA (Li, 2013). It then uses HiCExplorer (Ramírez *et al.*, 2018) to build and correct the Hi-C matrices using the iterative correction (ICE) method (Imakaev *et al.*, 2012). Matrices can be built at a user-specified resolution or at restriction fragment resolution by simply specifying the name of the restriction enzyme. The corrected HiC matrices can then be used for detection and visualization of topologically associated domains (TADs) (Dixon *et al.*, 2012). Quality reports are produced using HiCExplorer and are then summarized by MultiQC for comparison of samples.

The **WGBS** (whole-genome bisulfite-seq) workflow performs mapping of paired-end WGBS-seq data on a bisulfite-converted genome using bwa-meth (Pedersen *et al.*, 2014). To help assess the quality of the experiment, several metrics, including bisulfite conversion rate and coverage of random CpGs, are collected in a report. Counting of reads supporting methylated and unmethylated cytosines is performed with MethylDackel (https://github.com/dpryan79/MethylDackel). De novo discovery of differentially methylated regions (DMRs) can also be performed using Metilene (Jühling *et al.*, 2016) if a sample sheet is provided.

Processing of Online data

HiC, ChIP-Seq, and RNA-seq data for Smchd KO and wild-type Neural Progenitor Cells (NPCs) was downloaded from GSE99991. ATAC-Seq data for wild-type NPCs was downloaded from GSE71156 and WGBS data for wild-type NPCs was downloaded from GSE101090. All data was processed with snakePipes (version 1.0.0alpha5) on mouse genome GRCm38 (mm10).

The parameters specified for processing the data are as follows:

ATAC-Seq

DNA-mapping performed on mouse genome with parameters : `*-m allelic-mapping -j 30 --gcbias --mapq 5 --dedup --fastqc --trim --properpairs*

ATAC-seq performed on DNA-mapping output with parameters : `*--bw-binsize 10`.* By default, fragments longer than 150 nt are removed from peak calling `*--atac-fragment-cutoff 150*`.

ChIP-Seq

DNA-mapping performed on a dual hybrid (129S1/CAST) mouse genome with parameters : *`-m allelic-mapping --trim --fastqc --bw-binsize 10 --plotFormat pdf --dedup --mapq 10 --SNPfile <snp_positions.txt> --Nmasked_index <bowtie2_index.bt2>* `

ChIP-Seq performed on DNA-mapping output with parameters : `--bw-binsize 10` with *chip_sampleInfo.yaml* file which specified the corresponding input controls and peak-type. H3K4me3 samples were specified as `*sharp*` while the H3K27me3 samples were described as `*broad*`

RNA-Seq

RNA-seq workflow was run with parameters : `--fastqc --trim -m alignment,deepTools_qc --DE sampleinfo.tsv` where sampleinfo.tsv file defined groups with replicates (5 control and 5 knock-out samples). One of the knock-out samples was removed after inspecting PCA output (Fig. S1A) and workflow was re-run to obtain differentially expressed genes (Fig. S1B).

Hi-C

Hi-C workflow was run with parameters : `*--merge_samples --sampleInfo sampleinfo.tsv --distVsCount --bin_size 10000 --trim --fastqc*` where sampleinfo.tsv was used to define the two Hi-C replicates.

WGBS

WGBS workflow was run with all default parameters.

Output of online data processing

Below is the output folder structure after running snakePipes on the online data as mentioned above. The contents of each output folder are described in the online documentation for each workflow.

```
.
├── ATAC_seq
│   ├── allelic_bams
│   ├── ATAC-seq.cluster_config.yaml
│   ├── ATAC-seq.config.yaml
│   ├── pipeline.pdf
│   ├── ATAC-seq_run-7.log
│   ├── ATAC-seq_run-8.log
│   ├── atac_test.R
│   ├── bamCoverage
│   ├── Bowtie2
│   ├── cluster_logs
│   ├── CSAW
│   ├── deepTools_ATAC
│   ├── deepTools_qc
│   ├── diffATAC_sampeInfo.tsv
│   ├── DNA-mapping.cluster_config.yaml
│   ├── DNA-mapping.config.yaml
```

```
│   ├── dna-mapping.pdf
│   ├── FASTQ
│   ├── FastQC
│   ├── FastQC_trimmed
│   ├── FASTQ_Cutadapt
│   ├── filtered_bam
│   ├── filter_rules
│   ├── logs
│   ├── MACS2
│   ├── MACS2_QC
│   ├── multiQC
│   ├── Picard_qc
│   └── Sambamba
├── ChIP_seq
│   ├── bamCoverage
│   ├── Bowtie2
│   ├── chip_sampleinfo.yaml
│   ├── chip_samples.yaml
│   ├── ChIP-seq.cluster_config.yaml
│   ├── ChIP-seq.config.yaml
│   ├── ChIP-seq_pipeline.pdf
│   ├── ChIP-seq_run-1.log
│   ├── cluster_logs
│   ├── CSAW
│   ├── deepTools_ChIP
│   ├── deepTools_qc
│   ├── diffChIP_k27me3.tsv
│   ├── diffChIP_k4me3.tsv
│   ├── FASTQ
│   ├── FastQC
│   ├── FastQC_trimmed
│   ├── FASTQ_Cutadapt
│   ├── filtered_bam
│   ├── filter_rules
│   ├── histoneHMM
│   ├── logs
│   ├── MACS2
│   ├── multiQC
│   ├── Picard_qc
│   ├── QC_report
│   └── Sambamba
├── HiC
│   ├── BWA
```

```
│    ├── cluster_logs
│    ├── dist_vs_counts.png
│    ├── downstream
│    ├── FASTQ
│    ├── FastQC
│    ├── FastQC_trimmed
│    ├── FASTQ_Cutadapt
│    ├── HiC.cluster_config.yaml
│    ├── HiC.config.yaml
│    ├── HiC_matrices
│    ├── HiC_matrices_corrected
│    ├── HiC_pipeline.pdf
│    ├── HiC_run-1.log
│    ├── multiQC
│    ├── pipeline.pdf
│    ├── sampleinfo.txt
│    └── TADs
├── RNA_seq
│    ├── Annotation
│    ├── bamCoverage
│    ├── cluster_logs
│    ├── deepTools_qc
│    ├── DESeq2_sampleInfo_with_rep1
│    ├── DESeq2_sampleInfo_wo_rep1
│    ├── FASTQ
│    ├── FastQC
│    ├── FastQC_trimmed
│    ├── FASTQ_Cutadapt
│    ├── featureCounts
│    ├── logs
│    ├── multiQC
│    ├── QC_report
│    ├── RNA-seq.cluster_config.yaml
│    ├── RNA-seq.config.yaml
│    ├── RNA-seq_pipeline.pdf
│    ├── RNA-seq_run-1.log
│    ├── RNA-seq_run-2.log
│    ├── Sambamba
│    ├── sampleInfo_with_rep1.tsv
│    ├── sampleInfo_wo_rep1.tsv
│    └── STAR
└── WBGS
     ├── aux_files
```

```
├── bams
├── cluster_logs
├── FASTQ
├── FASTQ_Cutadapt
├── FASTQ_downsampled
├── logs
├── methXT
├── pipeline.log
├── QC_metrics
├── WGBS.cluster_config.yaml
├── WGBS.config.yaml
├── WGBS_pipeline.pdf
└── WGBS_run-1.log
```

Downstream analysis

We used the gene names DESeq2 output of snakePipes RNA-seq module `DESeq2_sampleInfo_wo_rep1/DEseq_basic_DEresults.tsv` using column 2 (basemean) and column 8 (Status) using awk (up-regulated : $8 == "UP", down-regulated: $8 == "DOWN", unchanged: $8 == "None" & $2 <= 10 | head -500) and subsetted the bed file `Annotation/genes.filtered.bed` for the entries using grep. For supplementary fig. 1d-e, we plotted ATAC-seq signal on these regions, using deepTools computeMatrix with options : *-a 5000 -b 5000* followed by plotHeatmap with option *--plotType se*. From WGBS workflow, we plotted the CpG methylation signal (bigWigs) on these regions using computeMatrix (--binsize 100) and plotHeatmap.

The results shown in figure 1b can be obtained by plotting the bigwigs and bed files from snakePipes output using pygenomeTracks, with these two commands:

make_tracks_file -o snakePipes_tracks.ini --trackFiles \
HiC/HiC_matrices_corrected/mergedSamples_NPC_WT_bs.corrected.h5 \
HiC/TADs/mergedSamples_NPC_WT_bs_domains.bed \
WGBS/NPC_WT_rep2_CpG.bw \
ATAC_seq/bamCoverage/WT_NPC_rep1.seq_depth_norm.bw \

```
ChIP_seq/deepTools_ChIP/bamCompare/allele_specific/NPC_WT_H3K4me3_rep1.genome1.lo
g2ratio.over_NPC_WT_Input_rep1.bw \
ChIP_seq/deepTools_ChIP/bamCompare/allele_specific/NPC_WT_H3K4me3_rep1.genome2.lo
g2ratio.over_NPC_WT_Input_rep1.bw \
ChIP_seq/deepTools_ChIP/bamCompare/allele_specific/NPC_Smchd1-KO_H3K4me3_rep1.ge
nome1.log2ratio.over_NPC_Smchd1-KO_Input_rep1.bw \
ChIP_seq/deepTools_ChIP/bamCompare/allele_specific/NPC_Smchd1-KO_H3K4me3_rep1.ge
nome2.log2ratio.over_NPC_Smchd1-KO_Input_rep1.bw \
ChIP_seq/deepTools_ChIP/bamCompare/NPC_WT_H3K27me3_rep1.filtered.subtract.NPC_W
T_Input_rep1.bw \
ChIP_seq/deepTools_ChIP/bamCompare/NPC_Smchd1-KO_H3K27me3_rep1.filtered.subtract.
NPC_Smchd1-KO_Input_rep1.bw \
RNA_seq/bamCoverage/NPC_WT_rep2.RPKM.bw \
RNA_seq/bamCoverage/NPC_Smchd1-KO_rep2.RPKM.bw \
RNA_seq/Annotation/genes.filtered.bed
```

```
pyGenomeTracks --tracks snakePipes_tracks.ini --region chrX:20423397-21530832 -out
schmd_tracks.pdf
```

Availability of data and materials

Online datasets re-analysed during this study are available in GEO with accession numbers : GSE99991, GSE71156 and GSE101090

Authors' contributions

VB developed the allele-specific and HiC workflows and contributed to DNA-mapping, ChIP-seq, ATAC-seq and RNA-seq workflows and documentation. SH developed the scRNA-seq workflow and documentation and contributed to DNA-mapping, ChIP-seq and RNA-seq workflows. DPR improved the wrapper design, integrated installation and conda support, contributed to the documentation and bug fixes to various workflows. KS developed the

WGBS workflow and documentation. LR contributed to HiC workflow and documentation. MR developed ATAC-seq workflow. FK contributed to RNA-seq workflow. AR contributed to DNA-mapping and ChIP-seq workflow. FK, SH and AR contributed to the general design of snakePipes and wrote the early version of the wrappers. VB performed the analysis and wrote the manuscript with input from all authors. TM conceived the project and supervised the development of snakePipes.

Competing interests

The authors declare no competing interests.

Supplementary References

Dixon,J.R. *et al.* (2012) Topological domains in mammalian genomes identified by analysis of chromatin interactions. *Nature*, **485**, 376–380.

Dobin,A. *et al.* (2013) STAR: ultrafast universal RNA-seq aligner. *Bioinformatics*, **29**, 15–21.

Ewels,P. *et al.* (2016) MultiQC: summarize analysis results for multiple tools and samples in a single report. *Bioinformatics*, **32**, 3047–3048.

Feng,J. *et al.* (2012) Identifying ChIP-seq enrichment using MACS. *Nat. Protoc.*, **7**, 1728–1740.

García-Alcalde,F. *et al.* (2012) Qualimap: evaluating next-generation sequencing alignment data. *Bioinformatics*, **28**, 2678–2679.

Hashimshony,T. *et al.* (2016) CEL-Seq2: sensitive highly-multiplexed single-cell RNA-Seq. *Genome Biol.*, **17**, 77.

Heinig,M. *et al.* (2015) histoneHMM: Differential analysis of histone modifications with broad genomic footprints. *BMC Bioinformatics*, **16**, 60.

Imakaev,M. *et al.* (2012) Iterative correction of Hi-C data reveals hallmarks of chromosome organization. *Nat. Methods*, **9**, 999–1003.

Jühling,F. *et al.* (2016) metilene: fast and sensitive calling of differentially methylated regions from bisulfite sequencing data. *Genome Res.*, **26**, 256–262.

Kim,D. *et al.* (2017) HISAT2: graph-based alignment of next-generation sequencing reads to a population of genomes.

Langmead,B. and Salzberg,S.L. (2012) Fast gapped-read alignment with Bowtie 2. *Nat. Methods*, **9**, 357–359.

Liao,Y. *et al.* (2014) featureCounts: an efficient general purpose program for assigning sequence reads to genomic features. *Bioinformatics*, **30**, 923–930.

Li,H. *et al.* 692 (2009). The Sequence Alignment/Map format and SAMtools. *Bioinformatics*, **25**, 2078–2693.

Li,H. (2013) Aligning sequence reads, clone sequences and assembly contigs with BWA-MEM. *arXiv [q-bio.GN]*.

Love,M.I. *et al.* (2014) Moderated estimation of fold change and dispersion for RNA-seq data with DESeq2. *Genome Biol.*, **15**, 550.

Lun,A.T.L. and Smyth,G.K. (2016) csaw: a Bioconductor package for differential binding analysis of ChIP-seq data using sliding windows. *Nucleic Acids Res.*, **44**, e45.

Martin,M. (2011) Cutadapt removes adapter sequences from high-throughput sequencing reads. *EMBnet.journal*, **17**, 10–12.

McKenna,A. *et al.* (2010) The Genome Analysis Toolkit: a MapReduce framework for analyzing next-generation DNA sequencing data. *Genome Res.*, **20**, 1297–1303.

Patro,R. *et al.* (2017) Salmon provides fast and bias-aware quantification of transcript expression. *Nat. Methods*, **14**, 417–419.

Pedersen,B.S. *et al.* (2014) Fast and accurate alignment of long bisulfite-seq reads. *arXiv [q-bio.GN]*.

Pimentel,H. *et al.* (2017) Differential analysis of RNA-seq incorporating quantification uncertainty. *Nat. Methods*, **14**, 687–690.

Ramírez,F. *et al.* (2016) deepTools2: a next generation web server for deep-sequencing data analysis. *Nucleic Acids Res.*, **44**, W160–5.

Ramírez,F. *et al.* (2018) High-resolution TADs reveal DNA sequences underlying genome organization in flies. *Nat. Commun.*, **9**, 189.

Tarasov,A. *et al.* (2015) Sambamba: fast processing of NGS alignment formats. *Bioinformatics*, **31**, 2032–2034.
