# Supplementary material for: snakePipes: facilitating flexible, scalable and integrative epigenomic analysis
Source: Bioinformatics. 2019 May 27;35(22):4757–9. doi: 10.1093/bioinformatics/btz436 (PMC6853707; doi:10.1093/bioinformatics/btz436)
Supplement: btz436_Supplementary_Data [file btz436_supplementary_data.zip › btz436-suppl_data/Supplementary_Table_1.pdf]

Supplementary Table 1				
**Criteria**	**snakePipes**	**Nextflow core pipelines**	**ENCODE-DCC**	**PiGx**
RNA-seq workflow	Released	Released	Under development	Released
DNA-seq workflow	Released	Not available	Not available	Not available
ChIP-seq workflow	Released	Under development	Released	Released
ATAC-seq workflow	Released	Not available	Released	Not available
scRNA-seq workflow	Released	Not available	Not available	Released
Whole-genome Bisulfite-seq workflow	Released	Released	Under development	Released
Hi-C workflow	Released	Not available	Under development	Not available
HLA-typing workflow	Not available	Released	Not available	Not available
Ancient DNA-analysis	Not available	Released	Not available	Not available
System dependencies	Conda and Python	Java8	Java 8 or higher, Cromwell, Docker CE	Guix, Autotools
Workflow language	snakemake	nextflow	WDL	snakemake
Installation	Conda	curl	Cromwell, dxWDL	Guix
Software dependency handling	Conda	Docker/Singularity/Conda	Docker or Conda	Guix, Conda
Parameter configuration	Editable yaml files; commandline arguments	Commandline arguments	json files	Editable yaml files, commadline arguments
Versioning	GitHub	GitHub	GitHub	GitHub
Cluster/resources configuration	Editable cluster.yaml files	Editable config file	.json and .conf files	Editable yaml files
Reference genome and index handling	createIndices workflow	Preconfigured links to iGenomes	Genome.tsv files are available on google cloud	needs to be setup by the user
Differential statistics on features between sample groups	Available	Not available	Not available	Available
QC report	MultiQC; workflow-specific QC; Html/Rmarkdown reports	MultiQC	workflow-specific QC	MultiQC, workflow specific QC, HTML reports
Test data bundle	Available via zenodo	GitHub	GitHub	GitHub
Documentation	Read the docs	Web; GitHub	GitHub	GitHub
Allele-specific mode	Available for most workflows	Not available	Not available	Not available
reference:	https://github.com/maxplanck-ie/snakepipes	https://github.com/nf-core	https://github.com/ENCODE-DCC/	https://github.com/BIMSBbioinfo/pigx
